# Patent term extension and test data protection obligations: identifying the gap in policy, research, and practice of implementing free trade agreements

**DOI:** 10.1093/jlb/lsad017

**Published:** 2023-07-18

**Authors:** Bryan Mercurio, Pratyush Nath Upreti

**Affiliations:** The Chinese University of Hong Kong, Shatin, New Territories, Hong Kong; School of Law, Queen’s University Belfast, Belfast, Northern Ireland

## Abstract

Much of the academic literature criticizes the inclusion of patent term extensions (PTE) and test data protection into the pharmaceutical provisions and/or intellectual property (IP) chapters of free trade agreements (FTAs), with many arguing that such provisions will increase the cost of pharmaceuticals for the implementing government. Such arguments are often backed by studies conducted prior to the conclusion of the relevant FTA. This is problematic for several reasons, most notably that the studies make assumptions that subsequently turn out not to be false and that the claims are not revisited and supported with empirical data following implementation. This article reviews the experience of two jurisdictions – Canada and Australia – in order to provide an analysis of legislative and judicial practices with a focus on implications and the cost of FTAs. The article examines how Canada and Australia have implemented their FTA obligations domestically and on the hereto ignored but important role of courts. One key finding is how courts in both countries are vigilant in narrowing the scope of obligations under FTAs to accommodate the need of the domestic market. The article ultimately concludes by calling on governments to conduct a detailed analysis of PTE and test data protection so as to better inform and prepare policymakers and, ultimately, improved FTA provisions and health outcomes.

## I. INTRODUCTION

Patent protection is essential to the innovative pharmaceutical industry as it provides incentives to engage in the research and development (R&D) of new products.^1^ To encourage and reward innovation and the advancement of science, patents provide the patent holder with the exclusive right to prevent third parties from making, using, offering for sale, selling, or importing for these purposes the patented product or process. Society needs a vibrant pharmaceutical industry; however, the scope of protection that the standard patent term can offer is limited. To this end, additional layers of protection have been added to domestic legislation and proliferated through the negotiation of Free Trade Agreements (FTAs).

The relationship between intellectual property rights (IPRs) and FTAs has received considerable attention in academic and policy discourse in recent years.^2^ Much of the discussion has focused on IP provisions in FTAs and their implication for public health and access to medicine. Two issues that have garnered much attention are the incorporation of patent term extension (PTE), and test data protection into FTAs.^3^ There is an abundance of existing literature criticizing the incorporation of PTE and test data protection (and other provisions that go beyond the minimum requirements established in the TRIPS) into FTAs, with much of the literature lamenting that the inclusion of TRIPS-Plus provisions undermine the minimum requirements established in the World Trade Organization’s (WTO) Agreement on Trade-Related Aspects of Intellectual Property Rights (TRIPS) with the assumption being that such provisions will have a negative effect on the price of pharmaceuticals and the sector more generally.^4^

These arguments are generally made in the abstract by scholars, non-governmental organizations, and sometimes by governmental bodies, often before the text of the relevant FTAs are finalized and sometimes once they initially become in force. Some of the claims estimate the potential implication of PTE and test data protection on drug prices if/when implemented in the domestic market of the country that signed the FTA. The claims are rarely revisited post-implementation nor is there an attempt to understand how the domestic implementation of the obligations and/or judicial interpretation of the domestic framework can impact the scope of PTE and test data protection.

This article addresses the gap through an examination of two jurisdictions—Canada and Australia—to understand how FTA obligations on PTE and test data protection are practiced and can be narrowed at the domestic level. More specifically, this article reviews the legislative and implementation practices of the two jurisdictions with a focus on implications and the cost of FTAs signed by those countries. These countries were selected as they share commonalities but also have some important differences. Most notably, while Canada has had test data protection in force for quite some time, it only recently introduced PTE following the negotiation of the European Union (EU)–Canada Comprehensive Economic and Trade Agreement (CETA).^5^ Conversely, Australia has had both test data protection and PTE for several years. While Canada has rich jurisprudence on test data protection and limited case law on PTE (due to the temporal limitation), Australia has clear jurisprudence on PTE but less case law on test data protection.

The main contribution of this article is to highlight the dearth of available economic analysis, generally on the impact of FTAs on pharmaceutical costs and in particular on the cost breakdown between and among test data protection and PTE. As will be demonstrated throughout this article, skeptics often publish ‘analysis’ prior to the conclusion of FTAs using inaccurate assumptions as to the make-up of the final text, whereas post-FTA studies tend to rely on macro-analysis and often fail to differentiate between causation and correlation. What is most surprising is that governments have shown little interest in conducting comprehensive studies or updating early and outdated models. While being a limitation, such information is also quite revealing and directly relevant to our conclusions and recommendations.

With this background in mind, the article proceeds as follows: after briefly providing a conceptual understanding of PTE and test data protection in Part II, Part III provides a background of legal instruments of test data protection and PTE in Canada, followed by an analysis of its practices under domestic law by virtue of Canadian obligations in FTAs. Part IV then discusses legislative practices of test data protection and PTE in Australia, followed by Australia’s FTA obligations through recent case laws. Part V concludes with the following key findings: first, studies that purport to show the impact of PTE and test data protection often make false assumptions, are outdated by the time they are published, and are not backed by sound empirical data; second, estimations on the impact of PTE and test data protection and indeed the literature more generally ignores the role that courts play in shaping (and narrowing) the scope of the obligations. Therefore, the impact of PTE and test data protection on the cost of pharmaceuticals remains inconclusive. We conclude by calling on governments to conduct a detailed analysis of PTE and test data protection so as to better inform and prepare policymakers and, ultimately, improve FTA provisions and health outcomes.

## II. PATENT TERM EXTENSION AND TEST DATA PROTECTION: A CONCEPTUAL OVERVIEW

### II.A. Patent Term Extension

The TRIPS Agreement requires WTO Members to grant patent protection for a period of at least 20 years from the date of the patent application, this being generally in line with the term of protection granted by large, industrialized countries prior to the advent of the TRIPS. The idea behind the set period of exclusive rights is clear—to allow inventors to recoup R&D costs and profit from the invention, thereby providing incentive for continued creation and scientific advancement. While the patent term is ordinarily 20 years from the date of filing a patent application, in regard to pharmaceuticals the effective patent term is significantly shorter due to delay resulting in the granting of the patent and/or from the health authorities in granting marketing approval for the pharmaceutical product. On average, it takes between 8 and 12 years to fulfill the requirements necessary to gain marketing approval for pharmaceutical products, meaning the effective patent term is on average 8 to 12 years.^6^

The PTE serves to compensate pharmaceutical companies/inventors for the loss during the period of regulatory delay. The most notable proponents of PTEs are the United States (US) and the EU, both of which were earlier adopters of the system (US in 1984 and the EU in 1992).^7^ Regionally, most of the more advanced economies have introduced PTE, including Australia (1999), Japan (1987), Singapore (2004), South Korea (2011), and Taiwan (2018). The structure of PTE differs among its adherents, but the intention behind PTE is shared—to provide a defined period of extended protection in order to counteract patent term erosion owing to regulatory delays as well as the expensive, complicated, and lengthy premarketing approval testing necessary in order to bring a new pharmaceutical product to market.

The PTE system is not explicitly recognized in multilateral treaties such as the TRIPS Agreement or the Paris Convention;^8^ instead, countries have introduced PTE on their own accord and as part of the intellectual property (IP) obligations in FTAs.

### II.B. Protection for Test Data

Protection for test data is *sui generis* right that can take two primary forms. Test data exclusivity protects data generated by the holder from being referred to or used by another person or company for a specific period of time. Test data exclusivity precludes the regulatory authority from even accepting applications from generic applicants relying on the test data until the end of the exclusivity period. By contrast, in what is referred to as ‘market exclusivity’, the regulatory authority can receive the application from the generic seeking to rely on the originator’s test data but it is prevented from granting marketing approval until the end of the exclusivity period. Test data exclusivity is the stronger form of protection, as it provides an additional period of de facto exclusivity equal to the time it takes the regulatory authority to consider the application and grant the marketing approval.

Test data protection is not a recognized IP right and can be categorized more as a quasi-IP right that attaches irrespective of whether the product is subject to patent protection. Test data protection provides the holder with limited-term protection against others using or referencing its preclinical, clinical trial or other data in an application for marketing approval or from the drug regulatory authority relying in its own right on the originator’s test data for approval of a generic pharmaceutical product. In this regard, test data protection is an automatic right and a negative right—it essentially acts as a right to exclude others from using as opposed to a right to use.^9^

It is important to reiterate that while test data protection prevents generic manufacturers from relying on innovator data to gain regulatory/marketing approval, a generic could generate its own test data by conducting clinical trials. This would, of course, add costs, and as clinical trials is not part of the generic business model and is arguably unethical as it would be repeating tests the results of which are already established. In this regard, test data protection acts as an incentive for firms to invest in and carry out clinical trials. It is also important to note that test data protection usually runs concurrently alongside patent protection, and often ends prior to the expiration of the relevant patents. Thus, any cost to test data protection occurs as a result of there being no original patent, the patent(s) being invalidated, or the expiration of the patent(s) prior to the conclusion of the test data protection. The latter occurs where, for instance, regulatory delays resulted in the drug coming to market with only a few years of patent protection remaining.

The objective of test data protection is to compensate the innovator/originator of a new product (and hence allow for the protection of their investment) for the time and money invested in inventing, testing, and bringing the product to market. The monetary costs involved in this process can be significant (not to mention the cost in time and human resources), and therefore it makes sense to ensure that the fruits of the investment are not simply handed over to generic competitors.

This need to incentivize clinical trials and the generation of test data is of course balanced by other interests, such as the right to health and information. Although the interpretation of Article 39(3) of the TRIPS Agreement is not settled, two distinct obligations could be inferred: Members must protect data against *unfair commercial use* and against *disclosure*, unless it is necessary to protect the public or unless steps are taken to protect against unfair commercial use. While the obligation against disclosure by the health authorities is relatively straightforward, the requirement to protect against ‘unfair commercial use’ is opaque. In what can only be termed ‘constructive ambiguity’, the drafters of the TRIPS provided no guidance or other information as to the meaning of the term. Thus, it is unclear how protection should occur, what the limit of such protection is or what the time period is for such protection. The question at the forefront of this debate is whether and to what extent Article 39(3) requires Members to preclude their drug regulatory authorities from using and relying on test data submitted by the originator in the examination of a generic product for a certain period of time.^10^

Numerous governments and commentators do not view the provision as requiring test data protection/exclusivity. ^11^ To them, there is nothing in Article 39(3) that requires WTO Members to prevent the health authorities from allowing generic applicants to rely on test data submitted by the originator in order to obtain marketing approval.^12^ Conversely, the US, EU, and a few others maintain that Article 39(3) mandates test data exclusivity.^13^

Test data protection is often included in the IP chapters of FTAs. The incorporation of test data protection in FTAs began in 1994 with Article 1711(6) of North American Free Trade Agreement (NAFTA) providing that test data submitted as part of the marketing approval process be protected and that ‘no person other than the person that submitted them may, without the latter’s permission, rely on such data in support of an application for product approval during a reasonable period of time after their submission’.^14^ The ‘reasonable period of time’ is defined as a period of 5 years.^15^ This approach subsequently served as a post-TRIPS template for test data exclusivity in FTAs.

## III. CANADA

### III.A. Background of Pharmaceutical Patent and Related Laws

Canada has a rich history of patents, with the Patent Act first coming into force in 1869. The landscape for pharmaceutical patents in Canada changed when the government amended the Patent Act in 1969 to permit compulsory licenses for the importation of medicines.^16^ By 1983, the change was estimated to have reduced the cost of pharmaceuticals in Canada by $211 million.^17^ At the same time, and despite losing market share, the innovative pharmaceutical industry was still profitable in Canada.^18^ Following these findings, the government introduced Bill C-22 (1987) to amend the licensing regime for patented medicines and to change the term of patent protection to 20 years from the filing date of a patent application.^19^ Though the amendments resulted in an increase in R&D spending in Canada, the government received criticism for restricting the issuance of compulsory licenses.^20^ During this period, Canada was also negotiating the Canada–US FTA and subsequently the NAFTA, which critics believe influenced the passage of Bill C-22.^21^

Subsequently, NAFTA led to an amendment of the Patent Act in 1992 severely restricting the issuance of compulsory licenses for medicines and the entry of generic producers into the Canadian market.^22^ In 1993, Canada also introduced ‘patent linkage’ provisions in the form of the *Patented Medicines (Notice of Compliance) Regulation*, which, among other things, required the Minister of Health not to issue Notice of Compliance (NOC) to generic companies until a patent is expired.^23^

### III.B. The Introduction and Experience with Test Data Protection

Like in other countries, innovator companies seeking regulatory approval for a new pharmaceutical product in Canada are required to submit the results of preclinical and clinical testing data to Health Canada to allow for verification that the product is safe and effective. Canada provided for test data protection in 2006 under the *Food and Drug Regulations* (the ‘Regulations’), as amended by the *Regulations Amending the Food and Drug Regulations (Data Protection), SOR/2006-241*.^24^ Enacted together with amendments to the *Patented Medicines (Notice of Compliance) Regulations*, the set of regulations were designed ‘to act as a balanced set of measures, designed to work together to stabilize Canada’s intellectual property protection for drugs by ensuring a minimum period of protection and maintaining a reasonable ceiling on the maximum protection available’.^25^

Prior to these regulations, a period of data protection exclusivity arose only when the Minister examined and relied on an innovator’s undisclosed data in order to grant a notice of compliance to a generic manufacturer. In practice, however, the Minister would normally assess the bioequivalence of a generic relying on the innovator’s product and without relying on the innovator’s data. According to the Regulatory Impact Analysis Statement (RIAS), which accompanies but does not form part of the regulations, the amendments were introduced ‘to clarify that the aforementioned reliance [on the innovator’s product] will give rise to an exclusivity period’.^26^

The Regulations provide innovative drugs with an 8-year term of data protection—submissions with pediatric studies are entitled to an additional 6 months of protection—thus preventing a another manufacturer from filing a submission for a generic version of that innovative drug for the first 6 years of the 8-year period.^27^ Data protection is conditional on the fulfillment of certain terms, namely that a drug has been given the approval to be added to Health Canada’s Register of Innovative Drugs^28^ at the time a NOC is issued to a manufacturer following the successful review of a submission for a new drug. Section C.08.004.1(3) of the Regulations is worth reproducing in full:

3) If a manufacturer seeks a notice of compliance for a new drug on the basis of a direct or indirect comparison between the new drug and an innovative drug,

(a) the manufacturer may not file a new drug submission, a supplement to a new drug submission, an abbreviated new drug submission or a supplement to an abbreviated new drug submission in respect of the new drug before the end of a period of six years after the day on which the first notice of compliance was issued to the innovator in respect of the innovative drug; and
(b) the Minister shall not approve that submission or supplement and shall not issue a notice of compliance in respect of the new drug before the end of a period of eight years after the day on which the first notice of compliance was issued to the innovator in respect of the innovative drug.^29^

Thus, the protection granted to innovative drugs under the data protection regulations takes two forms: (i) a generic drug manufacturer cannot file a submission based on a comparison to an ‘innovative drug’ within the first 6 years of the 8-year period after the drug has received a NOC;^30^ and (ii) the Minister cannot issue a NOC to the generic drug manufacturer before the end of the 8-year period.^31^ The Regulation continues to define an ‘innovative drug’ as ‘a drug that contains a medicinal ingredient not previously approved in a drug by the Minister [of Health] and that is not a variation of a previously approved medicinal ingredient such as a salt, ester, enantiomer, solvate or polymorph’.^32^

Canada’s 8-year base term of data protection is relatively weak when compared to others. While the term of protection applies to all drugs in Canada, jurisdictions such as the US and the EU provide additional years of protection for biologic drugs and in special circumstances—for instance, for orphan drugs in order to incentivize their R&D. Additional time and benefits are also available in most parts of innovative Asia, including Japan, Singapore, South Korea, and Taiwan. Here, Canada’s Orphan Drug Regulatory Framework stated that the 8-year data protection provisions under section C.08.004.1 of the Regulations will apply to market authorizations issued for orphan drugs^33^; however, data protection only applies to ‘innovative’ drugs and thus would exclude already known drugs that are further developed for orphan indications.

Moreover, Health Canada and Canadian courts have reduced the effectiveness of data protection by interpreting the regulations in a narrow manner and in such a way that has limited the legislation’s ability to stabilize Canada’s IP protection for drugs and serve as an incentive for Canadian innovation. The interpretations also call into question Canada’s conformity with its international obligations.

For example, decisions by the Federal Court of Canada (FCC) have determined and upheld a strict interpretation of the terms ‘previously approved’ and ‘variation’ in the definition of ‘innovative drug’. For instance, in the first case to consider the meaning of ‘innovative drug’ in light of a ‘previously approved’ drug,^34^ the court in *Epicept* rejected the challenge to the Minister’s decision that CEPLENE was not an ‘innovative’ drug. While CEPLENE was determined to be a ‘new drug’—it had been developed for a new oncology indication based on a new and full package of clinical data—its active ingredient (histamine dihydrochloride) was previously approved by the Minister for inclusion in another drug for homeopathic uses.

Notwithstanding the fact that homeopathics are not even approved in the same way as therapeutics and are subject to an entirely different Health Canada regulatory process, the FCC used the ‘previously approved’ criterion of the Regulations to uphold the Minister’s decision. Thus, the two points to take away from *Epicept* are as follows: (i) a ‘new drug’ for regulatory approval purposes will not necessarily be ‘innovative’ according to the Regulations; (ii) a new drug will be ineligible for data protection if its active ingredient has previously been given approval by way of another drug.

The FCC decision in *Celgene* likewise focused on the ‘previously approved’ aspect of an ‘innovative drug’, and centered on the Minister’s decision to refuse to list THALOMID on the Register of Innovative Drugs on the basis that its ingredient (thalidomide) had been approved decades earlier, despite the earlier drug having had another clinical usage and subsequently being withdrawn from the market for being unsafe.^35^ Arguing that it had generated its own clinical trial data for a separate disease and that the withdrawal of the unsafe drug in the 1960s was a relevant factor and nullified the previous approval, Celgene claimed THALOMID was in fact an ‘innovative’ drug. The FCC agreed, holding that the purpose and intent of the Regulations was to encourage innovation and the development of new drugs, and that THALOMID was an ‘innovative drug’. The Federal Court of Appeal (FCA), however, in a precedent setting case, reversed the decision and found that the word ‘approved’ is qualified by the adverb ‘previously’, and therefore any medicinal ingredient that previously satisfied Canadian regulatory requirements would be relevant, even if subsequently revoked.^36^

By contrast, the FCA in *Takeda* considered the basis for exclusion from data protection relating to the term ‘variations’.^37^ In this case, the court in a split decision rejected Takeda’s argument that the Minister erred in denying data protection for its drug DEXILANT, a drug used in the treatment of gastroesophageal reflux disease (‘GERD’), and an enantiomer (mirror image) of a previously approved drug also used in the treatment of GERD. Since dexlansoprazole was an enantiomer of a previously approved medicinal ingredient, the FCC upheld the Minister’s decision to deem it a ‘variation’ within the meaning of the Regulations and therefore not eligible for data protection.

The majority of the FCA agreed, holding that the five listed examples of variations in the definition of ‘innovative drug’ (a salt, ester, enantiomer, solvate, or polymorph), and therefore all enantiomers of previously approved medicinal ingredients, will always be a mere ‘variation’ and not qualify as ‘innovative drugs’. Writing for the Majority, Justice Dawson stated that while its obligations under NAFTA and TRIPS required that Canada consider what constitutes ‘new chemical entities’, but that it “…was open to the Governor in Council to decide, as a matter of policy, that salts, esters, enantiomers, solvates and polymorphs were not sufficiently different to be ‘new chemical entities’ and that the Court ‘…ought not to thwart the decision of the Governor in Council as expressed in the definition of ‘innovative drug””.^38^

Dissenting, Justice Stratas rejected a literal reading of the definition of ‘innovative drug’, finding instead that the term ‘such as’ meant that the definition was merely providing examples of substances which may be ‘variations’.^39^ To Justice Stratas, a drug should not be automatically excluded from protection solely on the basis that it contains an ingredient that is an enantiomer of a previously approved medicinal ingredient.^40^ The proper test should rather be whether the medicinal ingredients have ‘safety and efficacy characteristics materially different from a previously approved medicinal ingredient’.^41^ That such an ingredient might be an enantiomer would not therefore be determinative to the protection. In so finding, Justice Stratas also noted that the majority decision countered previous Minister decisions having granted data protection to certain drugs which contain medicinal ingredients that are esters or enantiomers, such as TORISEL, PRECEDEX, and AVAMYS^42^—and to point out that the main objective of the Regulations as per the RIAS is that ‘variations’ are excluded from the definition of ‘innovative drugs’ in order to prevent ‘the granting of an additional eight years of protection where an innovator seeks approval for a minor change to a drug’.^43^ Finally, the dissent expressed concern about the effect of undermining incentives for the development of beneficial new drugs.^44^

The Supreme Court of Canada refused Takeda’s application for leave,^45^ and the *Takeda* interpretation was subsequently applied in *Photocure*, where the FCC agreed with the Minister’s refusal to grant ‘innovative drug’ status to CYSVIEW because its active ingredient HAL HCl was a variation of ALA HCl.^46^ The important takeaway from this decision is that despite the FCA in *Takeda* applying a standard of review of the Minister’s interpretation of general correctness, the FCC reduced this to a lower threshold of reasonableness.

#### 1. Impact of the Restrictive Interpretation

The restrictive interpretation of the test data provisions has led to the perverse result of less drug availability in Canada.^47^ This can be illustrated in the *Horizon* case, where upon being rejected for test data protection by the Minister the applicant, Horizon, successfully moved to stay the issuance of its own approval pending judicial review of this decision.^48^ In the proceedings, Horizon stated that it would have to withdraw its drug RAVICTI to treat urea cycle disorders from the marketing approval process if it was not granted data protection in order to prevent generic competitors from using the information therein contained and entering the market immediately upon the expiry of its Canadian patent. The potential impact on industry and society was made clear: Horizon would suffer non-compensable losses and Canadian patients would be denied access to a life-changing and life-saving drug. In this case, the FCC reverted the decision to the Minister for reconsideration and RAVICTI was granted data protection. The reasons for the reconsideration were not made public. The same could not be said for Epicept, who upon being denied test data protection withdraw its submission for marketing approval.^49^

The denial of test data protection for drugs has been wide ranging and affects the treatments of life-threatening diseases such as cancer and leprosy, remission maintenance in acute myeloid leukemia, and the treatment of gastroesophageal reflux disease. The risk is innovators will not have sufficient incentives for product marketing approvals in Canada—which deny new treatments to patients afflicted with disease conditions. The issue is particularly acute with rare diseases, where the drugs at issue in the aforementioned cases have been denied protection in Canada but granted orphan drug status and associated benefits in other jurisdictions.^50^

Likewise, the restrictive interpretation of the law by the Minister and courts is at odds with other jurisdictions with a similar legal tradition. For instance, the US, Europe, Australia, New Zealand, and elsewhere do not deny data protection from drugs merely because their medicinal ingredients are enantiomers of previously approved drugs.^51^ The result, as demonstrated above, is that some innovative medical products will not be submitted to Health Canada and available for patients. This is not good for patients and also not good for generic drug manufacturers, which rely upon the clinical test data provided to Health Canada by innovators in order to proceed with the expedited generic drug approval process.

#### 2. Canadian FTA Obligations

As mentioned, Canada’s Section C.08.004.1 of the *Food and Drugs Regulation* adopts a period of 6 years of test data exclusivity and a period of 8 years of market exclusivity, and in so doing fulfills obligations under NAFTA. The recent update to NAFTA in the form of the United States–Mexico–Canada Agreement (USMCA) originally would have required parties to provide data protection for biologics for at least 10 years from the date of first marketing approval. However, amendments to USMCA removed this requirement for biologics.^52^ As a result, Canada maintained its existing test data protection laws that provide an 8-year term, with a possible 6-month extension.

Similarly, the EU–Canada CETA provides 8 years of data protection for chemical entities including a biologic or radiopharmaceutical ‘if the origination of such data involves considerable effort, except where the disclosure is necessary to protect the public or unless steps are taken to ensure that the data are protected against unfair commercial use’.^53^ Since Canada has placed 8 years of protection in place in its national system, no changes to the national law were needed to implement obligations arising from the CETA.

#### 3. Economic Effects of Test Data Protection

While many studies make sweeping generalizations on the cost of test data protection,^54^ the studies purporting to predict or account for the added costs of test data protection provisions in Canada are all flawed as they are based on assumptions that did not eventuate. This is even the case with the report by Canada’s Office of the Parliamentary Budget Officer, which not only assumed that enhanced data protection would incentivize the development of biologics but also that data protection would be the primary source of market exclusivity.^55^ The report further assumes that the USMCA would require 10 years of data protection for biologics, and therefore that biologics would cause additional expenditures.

The report assumed an annual increase in costs of $11.9 million and estimates that by 2029 there would be an additional expenditure of approximately $169 million, rising annually thereafter.^56^ While it is not rare for test data protection to exceed patent protection for biologic drugs, the Office of the Parliamentary Budget Officer also likely erred in estimating that biosimilar drugs would be offered at a 30 per cent cost savings and would account for 75 per cent of the market of the biologics losing data protection. Given the lack of biosimilar drugs on the market in Canada, these assumptions appear overstated. Using similar assumptions, another study concluded that extending data protection for 10 years would result in the cost of the drugs in Canada increasing by $200 million annually.^57^

Likewise, Lexchin and Gagnon updated a 2010 estimate and found that if CETA would require Canada to extend data exclusivity to what they deem but do not define to be ‘non-innovative drugs’, the average delay onto the market for generics would increase by 741 days (2.03 years) and result in an additional yearly cost of $1.645 billion, or 12.9 per cent of total costs of patented drugs.^58^ Bollyky provides a counter-point after evaluating FTAs with both PTE and test data protection from 2004 to 2013, finding that ‘drugs spending has remained flat as a share of overall health expenditure in countries with recent U.S. trade agreements’.^59^ Interestingly, Pham and Donovan looked at data from US FTAs between 2013 and 2018 and found a reduction in the average level of pharmaceutical expenditure.^60^ More recently, Brennan found a reduction in pharmaceutical expenditure in countries that have FTAs with EU.^61^ These studies indicate that FTAs with PTE or test data exclusivity do not *per se* impact drug pricing and lead to higher expenditures (nor did the findings indicate a reduction in access to innovative medicines).

Given that there are limited studies on the subject, the abovementioned projections cannot be fully relied upon to reach conclusions on the impacts of test data protection on drug pricing. While it is clear the narrow interpretation given to the legislation by the Minister and courts substantially reduce additional expenditures, it would be appropriate for the government to undertake a full review of the impact of test data protection on the cost of drugs to allow for informed policy decisions into the future.

### III.C. The Introduction and Experience with Patent Term Extensions

The Patented Medicines (Notice of Compliance) Regulations set out the relationship between Canada’s patent and pharmaceutical regulatory regimes.^62^ NOC Regulations provide a mechanism by which regulatory approval for a generic drug or subsequent entry biologics are operated. The data suggest that previously the delay for approval of new drugs by Health Canada took 527 days on average in 2010, which is longer than the approval process in the EU.^63^

Canada amended its Patent Act to create a framework for the certification of supplementary protection in 2017 in fulfillment of the terms of the CETA. As a result, Canada introduced the Certification of Supplementary Protection (CSP) Regulations, S.O.R./2017-165 to allow a form of PTE for pharmaceutical patents.^64^

The regulation provides CSP for up to 2 years of additional protection for eligible drugs as compensation for delays in obtaining market authorization. The patent’s eligibility for CSP depends on the subject matter of the claims. Section 2 of the CSP Regulations considers eligible patents only to be for products that contain a claim for the medicinal ingredient or combination of all the medicinal ingredients contained in a drug, even if all the medicinal ingredients are obtained by a specified process or use thereof.

#### 1. Judicial Interpretation

Until recently, the relevant provision of the CSP Regulations had not received any judicial interpretation. This changed in April 2020 with the decision in *GlaxoSmithKline Biologicals S.A. v Minister of Health*.^65^ In the case, the FCC interpreted the scope of the term ‘medicinal ingredient’. The issue reached the FCC following the Minister of Health’s refusal to issue a CSP with respect to Canadian Patent No. 26500,905 (905 Patent) and the drug SHINGRIX, a vaccine against shingles. The Minister refused to issue the CSP on the ground that the 905 patent did not fall under any eligible types of claims in Section 2 of the CSP Regulations—medicinal ingredient or combination of all the medicinal ingredients. Rather the Minister found that 905 patent claimed a formulation of an antigen (medicinal ingredient) and adjuvant (non-medicinal ingredient), which made the patent ineligible under the CSP Regulations.

The FCC reversed, finding the Minister’s decision unjustified on two grounds. First, the adjuvant used in the drug was biologically active and essential to its clinical efficacy, and therefore made the drug eligible for CSP application. Second, since the CSP Regulations does not define ‘medicinal ingredient’, the Federal Court referred to CETA to provide a purposive reading of the CSP Regulations. According to the Federal Court:

Although CETA defines an eligible patent (ie, a basic patent) as one that protects a ‘product as Such’, it also defines the protected product as ‘the active ingredient or combination of active ingredients’ in the approved drug or vaccine (CETA, Article 20.6). CETA does not refer at all to ‘medicinal ingredients’ and nowhere in Canadian legislation is that term defined.^66^

The plain reading of the preceding paragraph entails that the CSP Regulations must be read and interpreted in line with the relevant provision of CETA. To this end, the FCC was vocal on the harmonious interpretation of CSP Regulations with CETA:

The absence of any statutory definition for ‘medicinal ingredient’ is significant because the Canada-European Union Comprehensive Economic and Trade Agreement Implementation Act, SC 2017, c 6 [CETA Act] at section 3 directs that, unless otherwise stipulated, matters of statutory interpretation are to be resolved harmoniously with CETA…. To the extent that the applicable Canadian CSP legislation is open to interpretation, this provision calls for interpretive consistency with the language of CETA and not necessarily with Health Canada’s drug licensing guidelines.^67^

The FCC concluded that the application of the Minister’s position will narrow the scope of the CSP Regulations and ‘any new and useful vaccine that requires an adjuvant to be effective would be excluded for supplementary protection…. Inasmuch as many vaccines are adjuvanted and patented as such, this would exclude CSP protection for many novel vaccines’.^68^ Thus, the Federal Court believed the term ‘medicinal ingredient’ should not be interpreted too narrowly and must be read in accordance with the terms of the CETA.^69^

On appeal, however, the FCA found the Minister’s interpretation reasonable:

The Minister adopted a reasonable interpretation of the words ‘medicinal ingredient’ and made a scientific determination that in this case, the adjuvant was not in fact a medicinal ingredient because it had no independent therapeutic effect on the body; thus the Minister’s decision was based on a legal and scientific position backed up by the consistency between the medicinal ingredient listed in the NOC issued under the Food and Drug Regulations, the medicinal ingredient referred to in the application for a CSP and the Patent Act.^70^

Furthermore, the FCA found that the Minister’s reading of the CSP Regulations was not consistent with CETA. To this end, the FCA concluded that if the CSP Regulations are inconsistent with CETA, the issue should come before the CETA joint committee.^71^ Interestingly, while the court stressed that the proper question for the court is not whether the CSP Regulation is consistent with the CETA, Health Canada’s interpretation of the CSP Regulations was clearly based on Canada’s treaty obligations for pharmaceutical products. That being said, the court appeared concerned that interpreting the CSP regime with such a strong consideration to CETA might result in a broad interpretation of PTE, negatively affecting the entry of generic pharmaceuticals onto the market.

In July 2020, the Federal Court in *Natco Pharma (Canada) Inc v Canada (Health)* likewise seemed to be aware of the concern and emphasized that ‘an international treaty cannot be used to override the clear words of a statutory provision… [and the] treaty obligations cannot be considered independently of the words of the regulatory provision that implements them’.^72^ While this case did not concern PTE but rather whether the data protection provisions of the Food and Drug Regulations are triggered when an application for marketing approval is based on a comparison to a drug product that was in turn approved based on a comparison with an ‘innovative drug’, even though the direct comparator is not itself an innovative drug, it is nonetheless instructive as to the court’s position on the issue of treaty obligation.

In July 2020, the Federal Court in *ViiV Healthcare ULC v Canada* (Health) found the decision of the Minister denying a CSP in respect of Canadian Patent No. 2,606,282 (the 282 Patent) and the drug JULUCA unreasonable and remitted to the Minister for redetermination.^73^ In so deciding, the Court determined the Minister did not adequately consider ViiV’s submissions regarding the proper interpretation of the CSP regime in relation to CETA. In particular, the Minister relied on the CSP Regulations, RIAS and associated Guidance Document to offer an interpretation of Section 106(1) of the Patent Act without reference to the CETA;^74^ the Minister’s failure to consider whether the CSP Regulations could be read in harmony with CETA meant the Minister’s decision was ‘unreasonable’. Fuhrer J stated: ‘it is unreasonable for the Minister to rely only on these documents [CSP Regulation and RIAS] to the exclusion of CETA. The text of CETA, rather than, or in addition to, the CSP [Regulation] RIAS, also must be consulted for shedding light on and determining Canada’s intentions for the scope of protection applicable to Canada’s CSP regime.’^75^

While the Minister appeared to prefer a narrow interpretation to CSP, and argued that CETA signatories are ‘free to determine the appropriate method of implementing the provision of CETA within their own legal system and practice’.^76^ Fuhrer J disagreed:

In my view, a provision permitting signatories to implement given schemes in accordance with their own rules does not in itself absolve decision makers from adequately explaining that a more limited domestic interpretation was intended. There is nothing on the record in this matter to suggest Canada intended a more limited approach than what was contemplated in CETA…. Indeed, neither of these documents consider the text of CETA itself, nor the CIA, [CETA Implementation Act] when describing the intended scope of CSP protection. In such situations, the focus must be on what the legislator actually did in the legislation, not on what was said in such accompanying documents. Because domestic legislation is presumed to conform with a relevant treaty, the focus must be on what the legislator actually did in the legislation; this presumption requires the administrative decision-maker to take into account any relevant international law as part of the context surrounding the enactment of the legislation when interpreting it.^77^

With this decision, the Canadian Federal Court appears more inclined to adhere to a statutory interpretation approach that considers the text of the provision, the object of the Act and the intention of the Parliament. This is captured in the decision of the Supreme Court of Canada in *Canada (Minister of Citizenship and Immigration) v Vavilov* (2019), which holds:

[a] court interpreting a statutory provision does so by applying the ‘modern principle’ of statutory interpretation, that is, that the words of a statute must be read ‘in their entire context and in their grammatical and ordinary sense harmoniously with the scheme of the Act, the object of the Act, and the intention of Parliament’.^78^

Similarly, in *Canada Post Corp v Canadian Union of Postal Workers*, the Supreme Court of Canada maintained that ‘the administrative decision-maker must demonstrate in their reasons that they were alive to the essential elements of statutory interpretation’;^79^ such an approach reiterates the reading of *Canada (Minister of Citizenship and Immigration) v Vavilov.*

The jurisprudence on Canadian practice and experience with CSP is relatively new, and it is expected that it may take some years for there to be clear direction on whether the Canadian court’s interpretation of the CSP Regulations is broader than the European practice. What can be stated is that the Minister has been interpreting the CSP Regulations without considering the CETA, and in a narrow manner. Though the spirit of CETA provides freedom to determine the appropriate method of implementing the provision of CETA within own legal system, the Federal Court is more inclined to demand the CETA be considered and seems determined to take a wider interpretive approach. Perhaps this will eventually be sent to the Supreme Court of Canada for consideration.

#### 2. The Cost of CETA: Estimates and Reality

As of November 2022, 75 drugs had received a CSP (human use), with 12 refusals, 4 withdrawals, and 10 applications pending decisions.^80^ Most but not all CSPs have been issued for the full 2-year protection period. The number of CSPs will soon rise, as the Canadian Parliamentary Budget Office predicts that most of the drugs would be eligible for the full 2-year protection period.^81^ However, not all patents will be eligible for CSP protection.^82^ More specifically, Lexchin looked at those drugs listed in the Patent Register between January 1, 2008 to December 31, 2018,^83^ finding that the government report excluded 238 drugs (78 biologics and 160 small molecules) and that 218 (91.6 per cent) would meet the criteria of CSP with 192 (80.7 per cent) eligible for 2 years of protection.^84^ This shows that there is perhaps no clear estimate as to how many patents would be eligible for CSP protection in the near future and the length of protection that will be granted.

Several studies have mapped the potential impact of PTE and test data protection resulting from the CETA.^85^ Sibbald forecast that CETA’s pharmaceutical provisions are likely to increase Canadian drugs cost,^86^ echoing a study conducted by Lexchin and Gagnon that found that ‘full implementation of CETA would increase the average market exclusivity for patented drugs by 383 days, or 1.05 years, which would bring an additional yearly cost of least $850 million, or seven percent of total annual costs for patented drugs’.^87^ More specifically, Lexchin and Gagnon find the implementation of CETA will increase Canadian drug costs by between 6.2 per cent and 12.9 per cent starting in 2023.^88^ Likewise, Beall and others (2019) conclude that the legislative change in Canadian Pharmaceuticals post-CETA (including other FTAs) will increase national expenditure on patented drugs by $16 million and likely to result in an 11 per cent delay in generic entry—that is likely to result in annual cost more than $462 million depending on generic competitors.^89^ This has become more relevant as prices of generic drugs in Canada have dropped recently. Before the CETA, the average price unit of medicines increased for 12 percent year on year but have since dropped to 9.2 per cent per annum after CETA.^90^

Here again, however, the studies on the impact of CETA on Canada’s pharmaceutical regime both in terms of legislative change and impact of those changes are inaccurate as they are based on assumption that data exclusivity will be for 10 years, and that PTE will include a wider range of eligible drugs. Hence, the findings showing that CETA will impact drugs prices will not be accurate since Canada already provided for 8 years of data protection. The CETA will not impact the cost of test data protection in Canada in any way whatsoever.

Moreover, the impact of CSPs has not been felt and cannot accurately be predicted. To date, every CSP is to take effect in the future since the terms of CETA were not applied retroactively. That means that at this point there have not been any costs incurred due to CSPs because the original patents have not yet expired. In order to estimate the future cost of CSPs, the following information is needed: (i) the size of the market of every drug (ie, number of prescriptions and/or dollar sales) when the patent is due to expire; (ii) in order to know the market size in the future, one would also need to determine whether sales of the drug are increasing, stable, or decreasing; and (iii) how many generics will enter the market post-patent expiration since the number of generics and what share of the market the generics would capture determine the discount price that could be obtained.

## IV. AUSTRALIA

### IV.A. Background of Pharmaceutical Patent and Related Laws

The *Australian Patents Act 1990* allows for the grant of two types patent protection—standard patent and innovation patent. Standard patent protection is granted to any new and useful product, process, or machine that involves an inventive step that is made or used in industry. The standard patent lasts up to 20 years from the date of filing. By contrast, an innovation patent is granted on the basis of a lower standard and usually serves to protect an incremental invention that may have a reasonably short market life due to competition, but is nevertheless a new and useful improvement to existing inventions. In other words, an innovation patent does not meet the threshold of a standard patent but is a new innovation that has an incremental advantage over existing technology. The innovation patent lasts for a period of 8 years from the date of the filing.

As of August 26, 2021, the innovative patent is no longer available because the government opined that it did not achieve its intended objective and that it is often used by large firms as a tool to stifle competition.^91^ In addition, the *Therapeutic Goods Act 1989* and *National Health Act 1953* collectives provide a framework for incentivizing pharmaceutical innovations. The *Therapeutic Goods Act* read with its regulations guides the legal requirement for the import, export, manufacture, and supply of therapeutic goods in Australia. The Health Act, read with the framework of the Pharmaceutical Benefits Scheme (PBS), liaison the brand medicines and pricing.^92^

Pharmaceuticals constitute a large percentage of patent applications filings; between 1997 and 2011, 5.7 per cent of the patent application covered pharmaceutical inventions. A 2020 report conducted by IP Australia Indicates that the number has increased, with pharmaceutical patent applications rising by 6.5 per cent in 2019 and with overall year-on-year growth rate at 7 per cent—which is the highest compared to technology related to biotechnology, medical technology, civil engineering, and organic fine chemistry.^93^

### IV.B. The Introduction and Experience with Test Data Protection

#### 1. Legal Framework

The *Therapeutic Goods Act 1989 (Cth)* has since April 17, 1998 provided for a 5-year period of exclusivity for the use of information/data provided by a sponsor in an application for approval of a therapeutic good by the Therapeutic Goods Administration (TGA)—Australia’s governmental authority responsible for regulating therapeutic goods, including pharmaceutical products and medicines.^94^ Similar protections apply to data submitted in support of agricultural and veterinary products submitted for approval to the Australian Pesticides and Veterinary Medicines Authority under the *Agricultural and Veterinary Chemicals Code Act 1994 (Cth)*.

As with other jurisdictions, the period of data exclusivity is separate and distinct from other forms of IP protection, and can run concurrently with or extend beyond the IPR. Data protection remains in force and effective even if the IPR is rescinded or was never granted.

The data exclusivity provisions provide a defined period of time during which subsequent sponsors for equivalent products may not benefit from or rely upon the confidential data provided by the first sponsor (without their consent). In Australia, the period of exclusivity for data is 5 years from the date of marketing approval for products registered on the Australian Register of Therapeutic Goods (ARTG). The provision is limited to therapeutic goods containing a new ‘active component’, where no other therapeutic goods consisting of or containing that active component were included in the ARTG. An ‘active component’ is defined as a substance that is, or substances that together are, primarily responsible for the biological or other effect identifying the goods as therapeutic goods.^95^ The Explanatory Memorandum to the Act states that ‘substance’ may include ‘a biological product or compound’, which suggests that the data exclusivity period applies to biotechnology products requiring TGA approval. However, the protection is not offered to the listed therapeutic goods that represent the majority of complementary medicines.

The provisions only cover new active components that have never been included in the ARTG and do not encompass new uses of existing compounds. Data exclusivity is also not provided for new dosage forms, routes of administration, indications, or combinations with other active ingredients.

Data exclusivity in relation to an application to register an agricultural or veterinary product is provided for a period of up to 11 years, provided the application contains a new active constituent (ie, an active constituent that was not a previously endorsed active constituent at the time of registration), or a product containing a new active constituent, where that product has been accepted for evaluation before the active had been approved. Data protection is provided for a period of 5 years in relation to the approval to register another agricultural product, or registration variations in relation to an agricultural product, such as labeling. Data protection is provided for 3 years in relation to the approval to register another veterinary chemical product, or registration variations in relation to a veterinary chemical product, such as labeling.

The exclusivity periods apply to confidential information provided under the relevant legislation in support of the application for the registration of a product. The period of data exclusivity commences from the date on which the new product is registered under the TGA or Code, for pharmaceutical or agricultural and veterinary products, respectively.

Unlike in many other jurisdictions, test data protection is an automatic right; there is no application or assessment before it being granted. Thus, Australia does not have any procedural mechanism in the data protection regime to allow a generic or any other party to challenge the claim that information in the possession of the TGA is ‘protected information’ where the Secretary of the Department of Health takes the view that the information is ‘protected information’. On the other hand, if the Secretary of the Department of Health determines that information in the possession of the TGA is not protected information and proposes to use that information to evaluate a pending application for the registration of the pharmaceutical product on the ARTG, the originator (ie, the party that submitted the information to the TGA) may seek an injunction to prevent that use.^96^

#### 2. The Impact of Test Data Protection

The economic cost of the implementation of data exclusivity and the subsequent pharmaceutical import price inflation is difficult to analyze because of the lack of accurate information and evidence-based policy implementation.^97^ While numerous government and independent reports find cost increases and make forecasts for the future, none even attempt to ascertain the cost of test data protection.^98^ Prior to the implementation of the Australia–United States FTA, numerous articles estimated that Australia’s obligations would compromise the effectiveness of the PBS—for instance, Lokuge and Dennis predicted an increase in the national cost of pharmaceuticals of up to $2.4 billion.^99^ These studies, however, are either based on predicted changes that did not occur or on the basis of weak or no evidence. It is worth noting again that these forecasts did not break down the cost on the basis of IP-related provision (ie, test data protection, PTE, patent linkage, limits on compulsory licensing).

Rather curiously, two prominent Australian government reports (in 2013 and 2016, detailed below) did attempt to estimate the costs of PTE, but entirely ignored the economics of test data protection.

### IV.C. The Introduction and Experienc with Patent Term Extensions

#### 1. Legal Framework

The Australian Patents Act provides for the extension of the term of standard patents relating to pharmaceutical substances in order to compensate patent owners for the (sometimes significant) time it takes to obtain regulatory or marketing approval before sales of the related pharmaceutical substance can commence. Without the extension, a pharmaceutical patentee would almost always have a shorter effective term of patent protection to recoup the initial (and substantial) investment necessary to develop the pharmaceutical product. The objective behind the incorporation of PTE in Australia was also to attract investment in R&D in the pharmaceutical industry and make the Australian patent system competitive with other developed nations.^100^

Australia introduced PTE through amendments contained in the Intellectual Property Laws Amendment Act 1998,^101^ becoming effective on January 27, 1999. The amendments allowed for the extension of patent protection for a period of 5 years for pharmaceutical substances in order to offer a period of 15 years of ‘effective patent life’ for pharmaceutical products.^102^ From 1999 to 2014, nearly 95 per cent of the 697 applications for PTE were accepted, with more than half of all patents extended reaching or nearly meeting the 15-year effective life.^103^

Eligibility for the PTE is set out in Section 70 of the Patents Act. The criteria for the PTE application are:

1. The patent in substance discloses and claims: (i) one or more pharmaceutical substances *per se* must in substance be disclosed in the complete specification of the patent and in substance fall within the scope of the claim or claims of that specification, or (ii) one or more pharmaceutical substances when produced by a process that involves the use of recombinant DN technology, must in substance be disclosed in complete specification of the patent and in substance fall within the scope of the claim or claims of that specification.
2. Goods ‘containing, or consisting of’, the substance must be included in the Australian Register of Therapeutic Goods.
3. The first regulatory approval must be at least 5 years from the beginning of the date of the patent and the date on which the first regulatory approval for the pharmaceutical substance is issued.

In a nutshell, Section 70 of Patent Act allows for applications for one PTE (to be made within 6 months of the date of the patent or marketing approval, whichever is later) where at least a period of 5 years has elapsed between the period beginning on the date the patent was granted and ending on the first regulatory approval date for the substance and for those products where the term of patent has not been previously extended. The term of extension is equal to the period beginning on the date of the patent and ending on the first regulatory approval date of said pharmaceutical, reduced by 5 years, and not exceeding 5 years.^104^

Applications for a PTE must be made prior to the expiry of the term of the patent and within 6 months of the (i) date of grant of the patent or (ii) date of first regulatory approval of any goods containing the relevant pharmaceutical substance, whichever is later in time. When an extension of term is granted, the extension is be advertised for opposition purposes.

The PTE is equal to the time period between the filing date of the patent and the date of first regulatory approval minus 5 years. The extension of term period is capped at a maximum of 5 years. The rights afforded to the patentee during the extension period are somewhat more limited than in the normal patent period, as during the term of extension it is not considered an infringement to exploit the pharmaceutical substance for any purpose other than a therapeutic use or to exploit any form of the invention other than the ‘pharmaceutical substance per se’.

The extension is permitted only for patents that disclose and claim a ‘pharmaceutical substance’.^105^ Therefore, medical device or other medical products that have therapeutic effect are excluded from the eligibility of the patent extension. The assessment of qualification for the term extension depends on whether the patented pharmaceutical substance has received regulatory approval.

#### 2. Judicial Interpretation

The courts in Australia have considered, and debated, the meaning of ‘pharmaceutical substance per se’, including whether that meaning should be restricted to the active substance only or interpreted more broadly to include mixtures of substances, delivery systems comprising the active and the like. Reference to ‘goods containing, or consisting of, the substance’ in Section 70(3)(a) of Act has been interpreted narrowly such that even inclusion of minor amounts of the relevant substance—even as an impurity within the main active substance registration—is capable of starting the clock ticking on the application for extension deadline.

Over the years, the Australian courts have clarified the scope of the abovementioned criteria. In the *Boehringer Ingelheim International GmbH v Commissioner of Patents*,^106^ Heerey J. clarified the meaning of ‘pharmaceutical substances *per se*’, drawing a distinction between a pharmaceutical substance that is subject of a patent claim and a pharmaceutical substance that forms part of methods or process claim.^107^ This was done to clarify that ‘pharmaceutical substances *per se*’ refers only to pharmaceutical substance that is subject of a patent claim. Thus, method claims were excluded from the purview of the PTE. This reasoning was later endorsed in *Pre Jay-Holdings Ltd v Commissioner of Patents*.^108^

Another important requirement for the PTE is that goods ‘containing, or consisting of’ the substance must be included in the ARTG. In *Merck & Co., Inc v Arrow Pharmaceuticals Limited*, the court emphasized this criterion and stated that the assessment of the criteria is performed by comparing the pharmaceutical substance in question with the ‘active ingredients’ of the similar goods on the ARTG.

Two recent interpretive developments are noteworthy. The first is the case of *Commissioner of Patents v AbbVie Biotechnology Ltd*,^109^ which dealt with Section 70(2)(b), providing that an application for extension of term may be made where ‘one or more pharmaceutical substances when produced by a process that involves the use of recombinant DNA technology [are] disclosed in the complete specification of the patent and in substance fall within the scope of the claim or claims of that specification’. In the case, the Full Federal Court assessed whether extensions of patent terms are available for ‘Swiss-type’ claims (of a new use of a known substance) involving pharmaceuticals produced using recombinant DNA technology.^110^

The Deputy Commissioner of Patents had refused applications for a PTE on the basis of Swiss-type claims involving the therapeutic agent adalimumab, notwithstanding that adalimumab is typically produced by recombinant DNA technology (although this production method was not recited in the Swiss-type claims). The Administrative Appeals Tribunal of Australia reversed the decision, stating that the Swiss-type claims could allow for an extension of term, but the Full Federal Court reversed the Tribunal’s decision, reverting to the original finding of the Commissioner of Patents. In so doing, the Court stated that to make use of these provisions ‘the matter claimed must be the pharmaceutical substance or substances so produced, not other methods or processes involving those substances’. The Court went on:

The first and critical matter to note about Swiss type claims is that they are not claims to pharmaceutical substances at all. They are method or process claims which, in this connection, exhibit a dual character. First, they are directed to a method or process in which a substance is used to produce a medicament. Secondly, they have an additional method or process element constituted by a specific purpose to which the medication is to be used … the scope of Swiss type claims is fundamentally different to the scope of the claims addressed by s 70(2) of the Patents Act.^111^

Thus, Swiss-type claims (whether the substances recited are produced using recombinant DNA technology, as in this case, or otherwise) cannot be used to obtain a pharmaceutical extension of term. Moreover, the PTE must relate to claims to pharmaceutical substances, rather than related method claims. It seems unlikely therefore that method or process-type claims generally could be relied on for extensions of term under Section 70. Thus, a PTE will not be granted on the basis of a new use of a known therapeutic substance.

The second, more recent development is that the timing of the regulatory approval requirement has been subject to interpretation. In August 2021, *Ono Pharmaceutical Co, Ltd v Commissioner of Patents*,^112^ the Federal Court interpreted the ‘earliest first regulatory approval date’. The issue before the Court was the patent claims that encompassed two drugs that had received regulatory approval on different dates. Therefore, the question at issue was which regulatory approval date should be considered for the PTE request.

The patentee in this case simultaneously filed two requests for the PTE, one based on a competitor product that had received regulatory approval and the other on their own. The Patent Office refused the request to consider PTE based on their product (that would allow a longer extended term), concluding that the date when the competitor product received the earlier approval should be the basis for the PTE.^113^ However, the Federal Court of Australia reversed, concluding that the Patent Office’s interpretation was not ‘preferable’ and ‘leads to manifest absurdity or unreasonableness’.^114^ In so doing, the Court focused on the purpose of the PTE^115^ and concluded that if the construction of ‘earliest first regulatory approval date’ would also include a competitor ARTG registration, then there will be an unreasonable burden on the patentee to review each and every approval granted on the ARTG. In other words, every potential applicant would need to monitor regulatory approvals granted to third parties. The Court did not consider this reasonable, highlighting that a good faith search may fail to capture third-party products listed in the ARTG.^116^

Even more recently in *Merck Sharp & Dohme Corp v Sandoz Pty Ltd*,^117^ the Federal Court of Australia likewise analyzed the ‘earliest first regulatory approval date’ between two pharmaceutical substance of the patentee. Here, the Court concluded that the PTE must be based on the pharmaceutical substance that has the earliest regulatory approval date if both patented substance is of the same patentee. Unlike in *Ono Pharmaceutical Co*, the first regulatory approval date was not between the patentee and competitor or third party. This case, therefore, has greater relevance to the sector in general as it applies where a patent that covers more than one pharmaceutical substance.

One additional noteworthy development is the repeal of Section 76A,^118^ which required patentees to lodge to the Secretary of Health and Family Services a form setting out (a) details of the amount and origin of any Australian Commonwealth funds spent in the research and development of the drug that was the subject of the application; and (b) the name of anybody: (i) with which the applicant for the extension of term has a contractual agreement; and (ii) which is in receipt of Commonwealth funds; and (c) the total amount spent (in Australia) on each type of research and development, including pre-clinical research and clinical trials, in respect of the drug that was subject of the application. The section caused confusion among patentees, was not always followed or enforced, and was seemingly without rationale.

#### 3. The Impact of Patent Term Extensions

There is a perception that PTE is a cost to the system. For instance, a key finding of a panel set up in 2012 to review of pharmaceutical patents was that PTE did not increase R&D investment in pharmaceutical industries.^119^ Therefore, the panel recommended reducing the length of PTEs.^120^ Similarly, the Panel did not recommend PTE for orphan drugs, pediatric indications, and antibiotics.^121^

The Panel recommendation was considered in the Productivity Commission Report of 2016 as part of Australia’s Intellectual Property Arrangement,^122^ which drew a similar conclusion, calling the extensions of pharmaceutical patents ‘unwarranted’ and ‘expensive’ and suggesting PTE should only cover active pharmaceutical ingredients and be limited to specific instances of regulatory delay.^123^ The Panel reported that the annual cost to the PBS was estimated to grow from $6 million in 2001–2002 (shortly after the introduction of PTE) to $160 million in 2005–2006 ‘because there is a delayed entry to the PBS of cheaper generic drugs’.^124^ The Panel estimated the cost for 2012–20113 at around $240 million in the medium term and $480 million in the longer term.^125^ The Commission estimated the cost of PTE to Australia in 2016 to be $260 million per annum.^126^ In 2017, the Australian government responded to the Productivity Commission Report and ‘noted’ the recommendation made on PTE.^127^

## V. CONCLUSION

The cost of including test data protection and PTE is often reported to be high, but rigorous economic analysis is sparse, and often based on false assumptions and rarely attempts to isolate a particular form of protection (ie, test data protection from PTE). Another complication is the difficulty in accurately predicting the cost of extended exclusivity is how to take account of competition. Several studies fail to acknowledge the existence of therapeutic alternatives or equivalents or the emergence of new treatments that may reduce reliance on a pre-existing pharmaceutical product. Given these issues, it would be prudent to resist making assumptions that equate an additional duration of patent term or data exclusivity to the same volume of sales at the same price as occurred in earlier years.

Evidence that does exist is also contradictory, with at least one notable study finding no discernible increase in the price of pharmaceuticals when compared to the consumer price index for Canada or Australia from 2010 to 2019.^128^ For this reason, the true cost of each form of protection is often unknown and perhaps undiscoverable.

On the other side of the spectrum, there is also some evidence that FTAs with strong IP protection lead to substantial increases in investment and the absolute number of clinical R&D and trials conducted in countries incorporating strong IP protections—with one study claiming Australia and New Zealand would both benefit from stronger IP protections, which could result from an FTA with the EU and finding a 10.1 per cent increase in investment levels and increases between 70 and 80 per cent in clinical trial research in both Australia and New Zealand.^129^ This conclusion, however, is at odds with Australian government reports, which find that stronger IP protections have not led to greater investment in clinical R&D and trials. Here again, therefore, the economics of enhanced IP protection is uncertain.

This article focused on the patterns and discernible trends in Canada and Australia. In relation to test data protection in Canada, the ambiguous wording of the relevant text combined with a narrow interpretive approach used by the Minister has meant a refusal to grant protection for a wide range of pharmaceutical products. This has perhaps resulted in slight cost savings, but at the perverse cost of reduced access to life-saving drugs as originators withdraw applications for marketing approval where they are unlikely to profit from sales in the country. Given that Canada’s regime predates the CETA and did not include enhanced protection for biologic products, the CETA cannot be said to have added any additional cost to Canada’s health system in relation to test data protection.

In relation to PTE, the CETA will have an impact as Canada initiated a regime of providing CSP for up to 2 years of additional protection for eligible drugs as compensation for delays in obtaining market authorization only following commitments undertaken in the agreement. To date, there are no additional cost as the CETA did not apply retroactively and the patents remain in force for all CSPs. Emerging jurisprudence involves whether the Minister must consider the CETA commitments when considering whether to issue a PTE, with courts more inclined to answer the question in the affirmative.

In Australia, test data protection has been an automatic right since 1998, with no application or assessment before it being granted. The government has not conducted any studies on the economic impact of test data protection and few cases even touch on the subject. This is in contrast to PTE, where courts have taken an active role in shaping the substantive and temporal requirements for the grant of a PTE. The government also seems to have more interest in PTE with reports in 2013 and 2016 both criticizing the protection and putting figures on the cost to Australia (while also asserting that PTE has not led to increased investment in R&D in the country).

The two key findings of this article are therefore as follows. First, the estimation of an increase in drug prices in the domestic market as a result of FTA’s obligations of PTE and test data protection is inconclusive at best. As demonstrated throughout this article, there is a lack of empirical research to reaffirm the hypothesis that the FTA obligations of PTE and test data protection increase the cost of drugs in the domestic market. This is due to the fact that most estimates rely heavily on the assumption that an FTA with certain provisions could increase the price of drugs, but are not revisited to take account of the final text, implementation legislation, and judicial interpretations. To this end, the second finding of this article is that the domestic practice in Canada and Australia has shown how courts considerably narrow the scope of FTAs obligations. In other words, the courts play an important yet hereto unrecognized role in determining the scope of the FTA obligations of PTE and test data protection. The role of courts in determining the scope of FTA obligations of PTE and test data protection has not mapped out clearly in the literature; indeed, the current literature seemingly ignores the potential impact and implications of judicial interpretation.

Thus, we conclude that most of the discussion related to the impact of the FTA provisions of PTE and test data protection on the pricing of pharmaceutical products lacks solid empirical grounding. This is not to argue that one should assume the potential implications of PTE and test data protection obligations to be nil; rather, there is a need for governments to conduct a detailed analysis of PTE and test data protection so that policymakers can be better prepared and make better decisions leading to improved provisions in FTAs and health outcomes.

## Footnotes

1 Several studies point to the essential role of patents in promoting pharmaceutical innovation. See, eg, C.T. Taylor, A. Silberston and Z.A. Silberston, The Economic Impact of the Patent System: A Study of the British Experience 197–199 (1973); Edwin Mansfield, *Patents and Innovation: An Empirical Study*, 32(2) Management Science 173–181 (1986); Ashish Arora, Marco Ceccagnoli and Wesley M. Cohen, *R&D and the Patent Premium*, 26(5) International Journal of Industrial Organization 1172–1173 (2008); Brigitte Tenni et al, *What is the Impact of Intellectual Property Rules on Access to medicines? A Systematic Review*, 18(40) Globalization and Health 1–40 (2022); Iain Cockburn and Genia Long, *The Importance of Patents to Innovation: Updated Cross-Industry Comparisons with Biopharmaceuticals*, 25(7) Expert Opinion on Therapeutic Patents 739–742 (2015).

2 For several issues related to intellectual property and free trade agreements, see Intellectual Property & Free Trade Agreements, (Christopher Heath and Anselm Kamperman Sanders eds., 2007); Intellectual Property and Free Trade Agreements in the Asia-Pacific Region (Christoph Antons and Reto M. Hilty eds., 2015); EU Bilateral Trade Agreements and Intellectual Property: for Better or Worse? (Josef Drexl, Henning Grosse Ruse-Khan and Souheir Nadde-Phlix eds., 2014); Pratyush Nath Upreti, Intellectual Property Objectives in International Investment Agreements (2022) (discussing IP and dispute settlement mechanism under FTAs).

3
Ellen F.M.’T Hoen, The Global Politics of Pharmaceutical Monopoly Power: Drug Patents, Access, Innovation and the Application of the WTO Doha Declaration on Trips and Public Health (2009); Pepita Barlow et al., *The Health Impact of Trade and Investment Agreements: A Quantitative Systematic Review and Network Co-Citation Analysis* 13 Globalization and Health 1–9 (2017); Daniel Acquah, *Extending the Limits of Protection of Pharmaceutical Patents and Data Outside the EU — Is there a Need to Rebalance*, 45 International Review of Intellectual Property and Competition Law 256–286 (2014); Cynthia Ho, Access to Medicine in the Global Economy: International Agreements on Patents and Related Rights (2011).

4 See generally Wael Armouti and Mohammad Nsour, *Data Exclusivity for Pharmaceuticals in Free Trade Agreements: Models in Selected United States Free Trade Agreements*, 40 (1) Houston Journal of International Law 106–138 (2017); Piergiuseppe Pusceddu, *Assessing Access to Medicine in Preferential Trade Agreements: From the Trans-Pacific Partnership to the Comprehensive and Progressive Agreement for Trans-Pacific Partnership*, 49 International Review of Intellectual Property and Competition Law 1048–1079 (2018): Pratyush Nath Upreti, *From TPP to CPTPP: Why Intellectual Property Matters*, 13(2) Journal of Intellectual Property Law & Practice 100–101 (2018); Ruth Lopert and Deborah Gleeson, *The High Price of ‘Free’ Trade: U.S. Trade Agreements and Access to Medicines*, 41(1) Journal of Law, Medicine & Ethics 199–223 (2013).

5 Comprehensive Economic and Trade Agreement (CETA), https://policy.trade.ec.europa.eu/eu-trade-relationships-country-and-region/countries-and-regions/canada/eu-canada-agreement_en (accessed Dec. 10, 2022).

6 This often-cited figure can be found in, among other publications, European Commission, Dg Trade, Overview of the Pharmaceutical Sector in Europe (2011), https://www.hup.hr/EasyEdit/UserFiles/Granske_udruge/HUP-UPL/DG%20TRADE%20-%20OVERVIEW%20OF%20THE%20EU'S%20PHARMACEUTICALS%20SECTOR%20(16%20DECEMBER%202011).pdf (accessed Dec. 10, 2022).

7 For a historical record of the debate over term extension, see US Office Technology Assessment, Patent Term Extension and the Pharmaceutical Industry, NTIS order #PB82–100918 (1981), http://trade.ec.europa.eu/doclib/docs/2012/january/tradoc_148988.pdf (accessed Dec. 10, 2022).

8 Paris Convention for the Protection of Industrial Property (adopted 14 July 1967, entered into force 26 April 1970) 828 Unts 305.

9 See International Federation of Pharmaceutical Manufacturers and Associations (Ifpma), Encouragement of New Clinical Drug Development: The Role of Data Exclusivity 5 (2000), www.eldis.org/go/home&id=29224&type=Document#.WLpaTxKGNcA (accessed Dec. 10, 2022) (stating that test data exclusivity ‘provides a limited duration of time during which only the owner or generator of … preclinical and clinical trial data can *use* it for purposes of marketing authorization’).

10 TRIPS, Art 39(3) states ‘Members, when requiring, as a condition of approving the marketing of pharmaceutical or of agricultural chemical products which utilize new chemical entities, the submission of undisclosed test or other data, the origination of which involves a considerable effort, shall protect such data against unfair commercial use. In addition, Members shall protect such data against disclosure, except where necessary to protect the public, or unless steps are taken to ensure that the data are protected against unfair commercial use’.

11 For a review of the debate, see Bryan Mercurio, Drugs, Patents and Policy: A Contextual Study of Hong Kong Chapter 6 (2018)

12 See UNCTAD, *The Trips Agreement and Developing Countries*, UN, UNCTAD/ITE/11996 at 48, http://unctad.org/en/docs/ite1_en.pdf (accessed Dec. 10, 2022) (‘authorities are not prevented … from using knowledge of such data, for instance, to assess subsequent applications by third parties for the registration of similar products’).

13 See generally Bruce N. Kuhlik, The Assault on Pharmaceutical Intellectual Property, 71 University of Chicago Law Review 96 (2004); International Federation of Pharmaceutical Manufacturers and Associations (IFPMA), Data Exclusivity: Encouraging Development of New Medicines (July 2011), www.ifpma.org/wp-content/uploads/2016/01/IFPMA_2011_Data_Exclusivity__En_Web.pdf (accessed Dec. 10, 2022).

14 The North American Free Trade Agreement (NAFTA), signed Oct. 7, 1992, https://www.cbp.gov/trade/north-american-free-trade-agreement, Art 1711(6)

15
*Id*.

16 See *Patent Protection for Pharmaceutical Products in Canada–Chronology of Significant Events* (PRB 99-46E, prepared by Margaret Smith, Law and Government Division, Mar. 30, 2000), https://publications.gc.ca/collections/Collection-R/LoPBdP/BP/prb9946-e.htm (accessed Dec. 10, 2022).

17
*Report of the Commission of Inquiry on the Pharmaceutical Industry (Commission of Inquiry into the Pharmaceutical Industry*, prepared by Harry C. Eastman Commissioner, Feb. 28, 1985) xviii, https://publications.gc.ca/collections/collection_2016/bcp-pco/CP32-46-1985-1-eng.pdf (accessed Dec. 10, 2022).

18
*Id*.

19 Smith, *supra note* 16.

20 See Joel Lexchin, P*harmaceuticals, Patents, and Politics: Canada and Bill C-22*, 23 (1) International Journal of Health Services 147–160, 154 (1993).

21
*Id*.

22 Statutes of Canada 1993, C.2.

23 Patented Medicines (Notice of Compliance) Regulations, Mar. 12, 1993, SOR/93–133.

24
*Food and Drug Regulations*, C.R.C., c. 870.

25 Canada Gazette Part II: Regulatory Impact Analysis Statement, SOR/DORS/2006–241; 140(21):1495–1502 at 1499 [RIAS].

26
*Id*., at 1495–1496.

27 See Section C.08.004.1.

28 The Register of Innovative Drugs is available online: Health Canada, http://www.hc-sc.gc.ca/dhp-mps/prodpharma/applic-demande/regist/reg-innov-dr-eng.php (accessed Dec. 10, 2022).

29 Note that the Office of the Patented Medicines and Liaison (‘OPML’) makes the decisions on behalf of the Minister.

30 Subsection C.08.004.01(3)(a).

31 Subsection C.08.004.01(3)(b).

32 Subsection C.08.004.1(1).

33 Office of Legislative and Regulatory Modernization, Policy, Planning and International Affairs Directorate, Health Products and Food Branch, Initial Draft Discussion Document for a Canadian Orphan Drug Regulatory Framework, Orphanet, at J (Ottawa: Government of Canada, 2012), http://www.orpha.net/national/data/CA-EN/www/uploads/Initial-Draft-Discussion-Document-for-A-Canadian-Orphan-Drug--Regulatory-Framework.doc (accessed Dec. 10, 2022).

34
*Epicept Corporation v Canada (Health)*, 2010 F.C. 956 [*Epicept*].

35
*Celgene Inc. v Canada (Health)*, 2012 F.C. 154 [*Celgene*, FC].

36
*Canada (Health) v Celgene Inc*., 2013 F.C.A. 43 [*Celgene*, FCA].

37
*Takeda Canada Inc. v Canada (Health)*, 2013 F.C.A. 13 [*Takeda*].

38
*Takeda*, at 131.

39 Takeda, at 17.

40
*Id*.

41
*Takeda*, at 102.

42
*Takeda*, at 57–59.

43 RIAS at 1496 (cited in *Takeda*, at 63).

44
*Takeda*, at 98–102.

45
*Takeda Canada Inc. v Minister of Health, et al*., 2013 CanLII 33,948 (S.C.C.).

46
*Photocure ASA v Canada (Health)*, 2015 F.C. 959.

47 One exception should be noted; drugs that have been approved for emergency treatment under Health Canada’s Special Access Programme are not deemed to have been ‘previously approved’ as per the data protection regulations. See *Teva Canada Limited v Canada (Health)*, 2012 F.C.A. 106 (data protection maintained for ELOXATIN, which had been approved for emergency purposes in the absence of data and studies demonstrating its drug’s safety and efficacy).

48
*Horizon Pharma plc v Canada (Health)*, 2015 F.C. 744.

49 Epicept Corporation v. Canada (Health), 2011 F.C.A. 209.

50 CEPLENE has been granted orphan drug status for the treatment of acute myeloid leukemia by the European Medicines Agency and the U.S. Food and Drug Administration [Immune Pharmaceuticals, http://www.immunepharma.com/product-portfolio/ceplene/ (accessed Dec. 10, 2022); THALOMID has been granted orphan drug status in Europe for multiple myeloma and erythema nodosum leprosum [Evaluate, http://www.evaluategroup.com/Universal/View.aspx?type=Story&id=20950 (accessed Dec. 10, 2022); RAVICTI has been designated an orphan medicinal product in the EU for the treatment of urea cycle disorders [Horizon Pharma, http://ir.horizon-pharma.com/releasedetail.cfm?releaseid=944591 (accessed Dec. 10, 2022).

51 Justice Stratas made reference to the following examples of less restrictive legislation in *Takeda*, at 85: The Drug Price Competition and Patent Term Restoration Act, as amended by Pub. L. No. 110–85, 121 Stat. 823 (2007) (of the US); Regulation 726/2004 (of the EU); Therapeutic Goods Act, 1989, No. 21 (1990) (of Australia); and the Medicines Act, 1981, No. 118 (of New Zealand).

52 Protocol of Amendment to the Agreement Between Canada, the United States of America, and the United Mexican States, Dec. 10, 2019, https://www.international.gc.ca/trade-commerce/assets/pdfs/agreements-accords/cusma-aceum/protocol-amendment_protocole-amendement_eng.pdf (accessed Dec. 10, 2022).

53 CETA, art. 20.29 2(b), read with footnote 30.

54
European Public Health Alliance, Unhealthy Trades: The Side-Effects of the European Union’S Latin American Trade Agreements 20 (2018), https://epha.org/wp-content/uploads/2018/05/Unhealthy-Trade-Mercosur.pdf; Kenneth C. Shadlen, Bhaven N. Sampat and Amy Kapczynski, *Patents, Trade and Medicines: Past, Present and Future*, 27 Review of International Political Economy 75–97 (2020).

55
*The Impact of the Canada–United States–Mexico Agreement on Prescription Drug Expenditures in Canada* (Office of the Parliamentary Budget Officer, Apr. 2, 2019), https://www.pbodpb.gc.ca/web/default/files/Documents/Reports/2019/CUSMA/CUSMA_prescription_drug_expenditures_Canada_EN.pdf (accessed Dec. 10, 2022)

56
*Id*., at 22.

57 Dan Ciuriak, Ali Dadkhah, and Jingliang Xiao, *Quantifying the USMCA* (Paper Presented at the GTAP Annual Conference, Warsaw, June 2019) 23, https://www.gtap.agecon.purdue.edu/resources/download/9478.pdf (accessed Dec. 10, 2022).

58 Joel Lexchin and Marc-André Gagnon, *CETA and Pharmaceuticals: Impact of the Trade Agreement between Europe and Canada on the Costs of Prescription Drugs*, 10(30) Globalization and Health 1–6 (2014).

59 Thomas J. Bollyky, ‘A Dose of TPP’s Medicine: Why U.S. Trade Deals Have Not Exported U.S. Drug Prices’ (March 2016, Council on Foreign Relations Working Paper), https://papers.ssrn.com/sol3/papers.cfm?abstract_id=2755754 (accessed Dec. 10, 2022).

60 Nam D. Pham and Mary Donovan, *The Declining Trend of Pharmaceutical Expenditures in U.S. FTA Partner Countries*, NDP Analytics Study, June 10, 2019.

61 Orlaith Brennan, *The Impact of Pharmaceuticals IP Provisions in EU Free Trade Agreements–An Analysis Based on Real World Evidence* (May 2021) 22–23. This study also found a 182 per cent increase in pharmaceutical investments in Canada in the 3 years following the implementation of the CETA as compared to the 5 years preceding the agreement. *Id*., at 7.

62 Patented Medicines (Notice of Compliance) Regulations SOR/93–133.

63 Mark Rovere and Brett J. Skinner, *Access Delayed, Access Denied 2012: Waiting for New Medicines in Canada* (Studies in Health Policy, Fraser Institute, April 2012) (discussing data of 2010), https://www.fraserinstitute.org/sites/default/files/access-delayed-access-denied-2012.pdf (accessed Dec. 10, 2022).

64 See Certificate of Supplementary Protection Regulations, SOR/2017–165. See also Guide Document–Certificates of Supplementary Protection (Health Canada, Sept. 21, 2017).

65
*GlaxoSmithKline Biologicals S.A. v* Canada *(Health),* (2020 FCA 397).

66
*Id*., at para 26.

67
*Id*., at paras. 27 and 28 read together.

68
*Id*., at para 39.

69
*Id*.

70
*Canada (Health) v GlaxoSmithKline Biologicals S.A.,* (2021 FCA 71) para 70.

71
*Id*., at 117.

72
*Natco Pharma (Canada) Inc v Canada* (Health), 2020 FC 788, at para 52.

73
*ViiV Healthcare ULC v Canada (Health)*, 2020 FC 756.

74
*Id*., at paras. 14–15.

75
*Id*., para 26.

76
*Id*., para 21.

77
*Id*., para 27.

78
*Canada (Minister of Citizenship and Immigration) v Vavilov* (2019) SCC 65 para 117.

79
*Canada Post Corp v Canadian Union of Postal Workers*, 2019 SCC 67 at para 42.

80 See Certificates of Supplementary Protection (CSP) and Applications, https://www.canada.ca/en/health-canada/services/drugs-health-products/drugproducts/applications-submissions/guidance-documents/register-certificates.html (accessed Dec. 10, 2022).

81
*Patent Restoration and the Cost of Pharmaceuticals* (Office of the Parliamentary Budget Officer, Apr. 26, 2018); The Impact of the Canada-United States–Mexico Agreement on Prescription Drug Expenditures in Canada (Office of the Parliamentary Budget Officer, Apr. 2, 2019).

82 Joel Lexchin, *Development Time and Patent Extension for Prescription Drugs in Canada: A Cohort Study*, 10(8) International Journal of Health Policy Management 495–499 (2021).

83
*Id*., at 497.

84
*Id*., at 495.

85 See Lexchin and Gagnon, *supra note* 58; Joel Lexchin, *Increase in Drug Spending in Canada Due to Extension of Data Protection for Biologics: A Descriptive Study*, 14(3) Health Care Policy 10–18 (2019).

86 Barbara Sibbald, *Brand-Name Drug Costs Expected to Rise Under CETA*, 189(2) The Canadian Medical Association Journal E84-E85 (2017).

87 Lexchin and Gagnon, *supra note* 58 85.

88
*Id*., at 1.

89 Reed F Beall et al., *How Will Recent Trade Agreements that Extend Market Protections for Brand-Name Prescription Pharmaceuticals Impact Expenditures and Generic Access in Canada?* 123 (12) Health Policy 1251–1258 (2019).

90 See Brennan, *supra note* 61, 30.

91 Phase-out of the innovation patent (Australian Government, IP Australia), https://www.ipaustralia.gov.au/patents/applying-patent/innovation-patent-application-process/phase-out-innovation-patent (accessed Dec. 10, 2022).

92 See *Pharmaceuticals and Health* (Parliament of Australia), https://www.aph.gov.au/Parliamentary_Business/Committees/Senate/Former_Committees/freetrade/report/final/ch04 (accessed Dec. 10, 2022).

93 Australian Intellectual Property Report 2020 (Australian Government, IP Australia) p. 10 read with figure 4. https://www.ipaustralia.gov.au/sites/default/files/2020_ip_report.pdf (accessed Dec. 10, 2022).

94 Therapeutic Goods Act, Section 25A.

95 Therapeutic Goods Act, Section 25 A (3).

96 See ie, Alphapharm Pty Ltd v. H. Lundbeck A/S (2008) 76 IPR 618.

97 See generally Michael Palmedo, *Evaluating the Impact of Data Exclusivity on the Price per Kilogram of Pharmaceutical Imports* (Global Development Policy Center, GEGI Working Paper 48, 04/2021), https://www.bu.edu/gdp/files/2021/04/GEGI_WP_048_Palmedo_FIN.pdf (accessed Dec. 10, 2022).

98 See *Australia Pharmaceuticals Market–Growth, Trends, COVID-19 Impact, and Forecasts (2021–2026),* Mordor Intelligence, https://www.mordorintelligence.com/industry-reports/australia-pharmaceutical-market (accessed Dec. 10, 2022).

99 See Kamalini Lokuge and Richard Dennis, *Trading in Our Health System? The impact of the Australia-US Free Trade Agreement on the Pharmaceutical Benefits Scheme* (The Australia Institute Discussion Paper No 55, May 2003) 36, https://australiainstitute.org.au/wp-content/uploads/2020/12/DP55_8.pdf (accessed Dec. 10, 2022).

100 See the Explanatory Memorandum to the Intellectual Property Laws Amendment Bill 1997, pp. 3–7 and 9 read together.

101 Intellectual Property Laws Amendment Act 1998, section 3, Sch.1.

102 The Australian Patents Act 1990, Section 70. See also, Explanatory Memorandum to the Intellectual Property Laws Amendment Bill 1997 p. 4.

103 Productivity Commission Report 2016, p 293, https://www.pc.gov.au/inquiries/completed/intellectual-property/report (accessed Dec. 10, 2022).

104 The Australian Patents Act 1990, Arts. 70, 71, and 77 of the Patent Act 1990.

105 The Australian Patents Act 1990, Section 70 (2).

106
*Boehringer Ingelheim International GmbH v Commissioner of Patents*, (2001) AIPC 91–670.

107 The meaning of pharmaceutical substance per se was also discussed in Alphapharm Pty Ltd v. H Lundbeck A/S (2005) FCAFC 138; *H Lundbeck A/S v Alphapharm Pty Ltd* (2009) FCAFC 70.

108
*Pre Jay Holdings Ltd v Commissioner of Patents* (2002) FCA 881, at para 13.

109
*Commissioner of Patents v AbbVie Biotechnology Ltd* [2017] FCAFC 129.

110 The general form of a Swiss-type claim can be exemplified as ‘*Use of substance X in the manufacture of a medicament for the treatment of condition Y*’. Swiss-type claims are interpreted as use-limited under Australian practice, such that a Swiss-type claim involving the use of a known ‘*substance X*’ will be considered novel if the treatment of ‘*condition Y*’ with the known substance has not been previously described.

111 See also *Otsuka Pharmaceutical Co., Ltd v Generic Health Pty Ltd (No 4) [2015] FCA 634.*

112
*Ono Pharmaceutical Co, Ltd v Commissioner of Patents (2021) FCA 643 (June 11, 2021)* [*Ono Pharmaceutical v Commissioner of Patents*].

113 Ono Pharmaceutical Co., Ltd, et al (2020) APO 43.

114
*Id*., para 182.

115
*Id*., para 59.

116
*Id*., paras. 162–172, 162–163.

117
*Merck Sharp & Dohme Corp v Sandoz Pty Ltd*, (2021) FCA 947.

118 Intellectual Property Laws Amendment (Productivity Commission Response Part 1 and Other Measures) Act 2018, commencing on Aug. 25, 2018.

119 Pharmaceutical Patents Review Report, 2013, https://www.ipaustralia.gov.au/sites/default/files/2013-05-27_ppr_final_report.pdf (accessed Dec. 10, 2022).

120
*Id*., Recommendation 4.1.

121
*Id*., 88–89.

122 Productivity Commission Report 2016, https://www.pc.gov.au/inquiries/completed/intellectual-property/report (accessed Dec. 10, 2022).

123
*Id*., 297.

124 Pharmaceutical Patents Review Report, 2013, p vii–viii, 63 (citing the Revised Explanatory Memorandum to the Intellectual Property Laws Amendment Bill 1998, p 2).

125
*Id.*

126 Productivity Commission Report 2016, *supra note*122, 298.

127 Australian Government Response to the Productivity Commission Inquiry into Intellectual Property Arrangements, August 2017, https://www.pc.gov.au/inquiries/completed/intellectual-property/intellectual-property-government-response.pdf (accessed Dec. 10, 2022).

128 See Brennan, *supra note* 61, 24.

129
*Id*., 8.

